# Assessment of quality of life in lung cancer patients undergoing chemotherapy

**DOI:** 10.1192/j.eurpsy.2025.1771

**Published:** 2025-08-26

**Authors:** M. Abdelkefi, I. Gassara, R. Jbir, R. Feki, B. Abderrahmane, R. Khemakhem, N. Charfi, N. Smaoui, S. Omri, J. Ben Thabet, M. Maalej, S. Kammoun, M. Maalej, L. Zouari

**Affiliations:** 1 Psychiatry C department; 2 Department of pneumology, Hedi Chaker university hospital, Sfax, Tunisia

## Abstract

**Introduction:**

Lung cancer remains one of the leading causes of cancer-related morbidity and mortality worldwide. Despite being a crucial treatment, chemotherapy often brings a range of side effects that can significantly impact the overall quality of life of the patients.

**Objectives:**

To evaluate the quality of life (QoL) in lung cancer patients undergoing chemotherapy.

**Methods:**

The sample consisted of 49 lung cancer patients undergoing chemotherapy at the Pneumology and Allergology Department of the Hedi Chaker University Hospital in Sfax. The questionnaire used included patients’ sociodemographic characteristics, cancer and treatment data, and the European Organization for Research and Treatment of Cancer Core Quality of Life Questionnaire 30-item version (EORTC QLQ-C30).

**Results:**

The mean age of the patients surveyed was 61,8 years, with a male predominance (85,7%). The most frequently reported functional signs of lung cancer were dyspnea (36,7%), cough (34,7%) and chest pain (20,4%). Disease duration was less than 1 year in 61,2% of the cases, and 59,2% were classified as stage IV. Tumor progression occurred in 32,7% of cases. Of the patients, 20,4% were on a single chemotherapy agent, while 79.6% were on a combination regimen.

According to QLQ-C30, the mean global QoL score was 61,24 ± 24,5 for the entire sample. Physical functioning and role functioning were the most affected on the functional scale, while fatigue and appetite loss were the most frequent symptoms.

**Conclusions:**

The findings indicate that lung cancer patients undergoing chemotherapy experience significant challenges to their quality of life, particularly in physical functioning and symptom management. These results highlight the critical need for integrating routine QoL assessments into clinical practice to better address patients’ needs and improve supportive care.

**Disclosure of Interest:**

None Declared

